# Generating single metalloprotein crystals in well-defined redox states: electrochemical control combined with infrared imaging of a NiFe hydrogenase crystal†
†Electronic supplementary information (ESI) available: Crystal preparation; cyclic voltammogram in the experimental cell; position-dependent difference spectra; demonstration of control over all active sites; visible images of a crystal recorded periodically during measurements; time-dependent Ni_a_-R formation. See DOI: 10.1039/c7cc02591b


**DOI:** 10.1039/c7cc02591b

**Published:** 2017-05-09

**Authors:** P. A. Ash, S. B. Carr, H. A. Reeve, A. Skorupskaitė, J. S. Rowbotham, R. Shutt, M. D. Frogley, R. M. Evans, G. Cinque, F. A. Armstrong, K. A. Vincent

**Affiliations:** a Department of Chemistry , University of Oxford, Inorganic Chemistry Laboratory , South Parks Road , Oxford , OX1 3QR , UK . Email: kylie.vincent@chem.ox.ac.uk; b Research Complex at Harwell , Rutherford Appleton Laboratory , Didcot , Oxfordshire OX11 0FA , UK; c Department of Biochemistry , University of Oxford , South Parks Road , Oxford , OX1 3QU , UK; d Diamond Light Source , Harwell Science and Innovation Campus , Didcot , Oxfordshire OX11 0QX , UK

## Abstract

We describe an approach to generating and verifying well-defined redox states in metalloprotein single crystals by combining electrochemical control with synchrotron infrared microspectroscopic imaging. For NiFe hydrogenase 1 from *Escherichia coli* we demonstrate fully reversible and uniform electrochemical reduction from the oxidised inactive to the fully reduced state, and temporally resolve steps during this reduction.

## Abstract 2

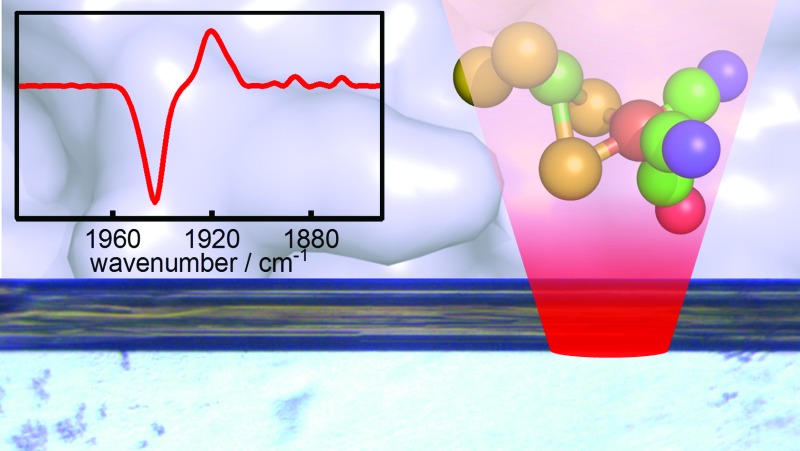
We manipulate and verify the redox state of single metalloprotein crystals by combining electrochemical control with synchrotron infrared microspectroscopy.

## 

Protein X-ray crystallography is well-established for contributing to structural understanding of the mechanisms of redox metalloproteins, but significant challenges remain in how to prepare protein crystals in well-defined functionally-relevant states.^1^ Present approaches include conducting multiple crystallisations with the protein poised at a range of redox levels, or infusing pre-formed crystals with reductants or oxidants in solution. However, there is often no means of directly controlling and verifying the state or uniformity of the crystallised sample prior to recording the diffraction pattern. Crystallographic structures reported for metalloproteins can represent structural averages from mixtures of states at similar redox levels. Further concerns arise from the inevitable X-ray induced reduction of metal ions during crystallographic data collection.^2^,^3^ In order to gain a more satisfactory understanding of metalloprotein function, complementary microspectroscopic techniques must be used. Whilst optical techniques have been applied to on- and off-line monitoring of single protein crystals,^4^,^5^ methods which offer direct control over the oxidation state of the crystal are lacking. Here we demonstrate a new method that combines precise electrochemical control over the redox state of a crystallised protein with *in situ* infrared (IR) microspectroscopic imaging.

We demonstrate this approach using single crystals of NiFe hydrogenase 1 (Hyd1) from *Escherichia coli* which crystallises readily to yield large, well-diffracting crystals.^6^,^7^ In addition Hyd1 is an important biocatalyst for oxidation of H_2_,^8^,^9^ and is a member of the ‘O_2_-tolerant’ NiFe hydrogenases.^7^,^10^,^11^ NiFe hydrogenases possess biologically unusual CO and CN^–^ ligands coordinating the iron atom at their active site, and vibrational stretching bands in the IR spectrum arising from these ligands (*ν*_CO_ and *ν*_CN_ respectively) provide a sensitive probe for the redox state of the active site. In this way, a number of redox levels of the NiFe site have been characterised by applying IR spectroscopy or spectroelectrochemistry to the enzymes in solution,^10^–^12^ or immobilised on a carbon electrode in the approach termed protein film infrared electrochemistry, PFIRE.^13^,^14^ Scheme 1 shows the redox states of the Hyd1 active site relevant to this work.^13^,^15^ The most oxidised state of the active site is the catalytically inactive Ni–B state containing a deprotonated water molecule between the metals. Reductive activation produces Ni_a_–SI with a vacant bridging site that becomes occupied by a bridging hydride upon proton-coupled reduction to the Ni_a_–C or Ni_a_–R levels.^10^,^16^–^18^ (Ni_a_–SI is also the state which reacts with H_2_ to initiate the H_2_ oxidation catalytic cycle.^19^) In Hyd1, Ni_a_–C is in tautomeric equilibrium with Ni_a_–L, in which the hydride has moved as a proton to a nearby basic site. Ni_a_–R and Ni_a_–L each represent a collection of up to three sub-states which are thought to differ in protonation close to the metal centre.^12^,^20^ We show here that it is possible to electrochemically navigate between these states in a single crystal, and furthermore, that retarded reactivity in the crystal permits resolution of the kinetics of formation of individual Ni_a_–R sub-states, which otherwise appear simultaneously in solution.

**Scheme 1:**
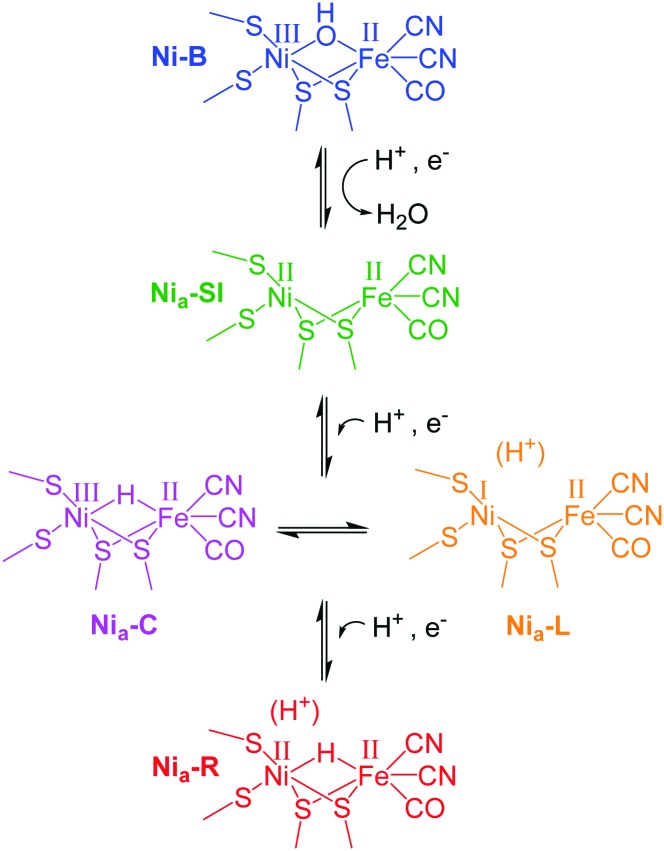
Active site redox levels of Hyd1 relevant to this study. Catalytically active states are labelled ‘Ni_a_–X’, where X = SI, C, R or L.

A number of groups have reported IR microspectroscopy cells for measurements under electrochemical control.^21^–^25^ Here, we report an adaptation of our previous cell design^25^ for experiments with single crystals of Hyd1, where the crystal is deposited directly on a 1 mm diameter glassy carbon (Alfa Aesar) working electrode (WE, Fig. 1). The electrochemical cell contained a 2 mm diameter saturated calomel reference electrode (SCE RE) constructed as described previously,^13^ and a removable Pt wire counter electrode (CE) ring surrounding the working electrode. Potentials (*E*) were converted to volts (*V*) *vs.* the standard hydrogen electrode (SHE) using the conversion *E*(SHE) = *E*(SCE) + 241 mV.^26^ The working electrode was polished prior to crystal loading using increasingly fine grades of silicon carbide paper (to 4000 grit, Kemet). The polished electrodes were then washed by ultrasonication in ultrapure water.

**Fig. 1:**
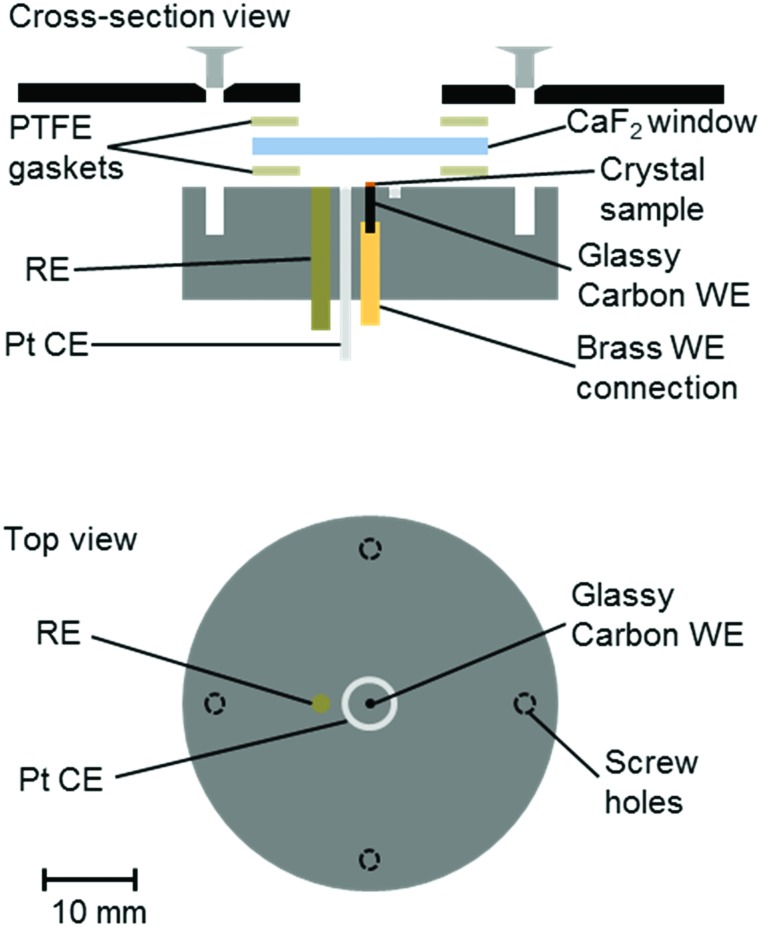
Schematic representation of the infrared microspectroscopic-electrochemical cell showing the placement of the crystal sample directly onto a glassy carbon working electrode (WE), and positioning of the reference and counter electrodes (RE, CE). Approximately to scale, as indicated.

Crystals of Hyd1 were manually harvested using a cryo-loop (Hampton Research) and transferred into a 5–10 μL droplet of crystallisation buffer on the working electrode with the aid of an optical microscope (see ESI†). An additional 2–3 μL aliquot of crystallisation buffer, containing the redox mediators methylene blue, methyl viologen and 2-hydroxy-1,4-naphthoquinone (Sigma, each at 5 mM concentration) was pipetted over the crystal on the working electrode. The crystallisation buffer therefore served as electrolyte, containing a final concentration of 1–3 mM of each mediator. The redox mediators diffuse throughout the microspectroscopic-electrochemical cell and facilitate electron transfer between the working electrode and the crystalline Hyd1. A CaF_2_ window (Crystran, 25 mm diameter, 2 mm thickness) was then placed over the crystal and the cell was sealed using two PTFE gaskets (Harrick, 12 μm). Microspectroscopy experiments were carried out on the MIRIAM beamline at the Diamond Light Source, UK, using a Vertex 80V FT-IR spectrometer and a Hyperion 3000 IR microscope (Bruker) with a high-sensitivity photovoltaic mercury cadmium telluride (MCT) detector cooled to 77 K with liquid N_2_. Spectra were acquired in reflection mode (hence each spectrum results from IR radiation passing through the thickness of the crystal twice) using a 36× objective and 15 × 15 μm^2^ aperture, as an average of 1024 interferograms at 4 cm^–1^ resolution and 40 kHz interferometer frequency. Data acquisition was performed using Bruker Optik's OPUS software (version 7.0), and data analysis was carried out using OriginPro software (OriginLab). Electrochemical control was achieved using an Autolab 128N potentiostat (EcoChemie) controlled by Nova software (version 1.10). A representative cyclic voltammogram of the crystallisation buffer containing redox mediators is shown in Fig. S1 of the ESI.†

Crystals of Hyd1 are up to 50 × 50 μm^2^ in cross section and 1000–2000 μm in length (the crystals used in this work are somewhat smaller, approximately 10 × 22 μm^2^ in cross section, Fig. 2A), allowing spectra to be collected on sub-sections of the crystal. Fig. 2A shows a visible image, at two magnifications, of a single Hyd1 crystal lying on the glassy carbon working electrode (fine scratches are also visible in the glassy carbon surface). Black squares show the 15 × 15 μm^2^ area used to record the IR spectra reported in Fig. 2B. NiFe hydrogenases are typically isolated in a range of oxidised inactive states which can be reductively activated at different rates.^10^ Crystals prepared aerobically from ‘as-isolated’ Hyd1 contain Ni–B (Scheme 1) and an alternative oxidised inactive state termed Ni–A which is defined by oxidative damage to Cys-79, one of the ligands coordinated to the nickel ion in the active site.^6^,^27^ Analysis of electron density maps suggest levels of Ni–A to be between 30 and 50%, depending on the age of the protein crystal prior to freezing. The Ni–A and Ni–B states are indistinguishable using IR spectroscopy alone due to the similarity of their *ν*_CO_ vibrational bands.^10^–^12^ Therefore in order to remove Ni–A, the Hyd1 crystal was first subjected to reductive activation for 8.5 hours at –359 mV, and the potential was then stepped back to +241 mV to re-oxidise the enzyme. This generated a sample entirely in the fully oxidised Ni–B state.^28^ The low potential poise at –359 mV also serves to reduce any O_2_ present in the crystallisation buffer electrolyte solution; since the cell is sealed with a PTFE gasket the remainder of the experiment is then conducted under anaerobic conditions.

**Fig. 2:**
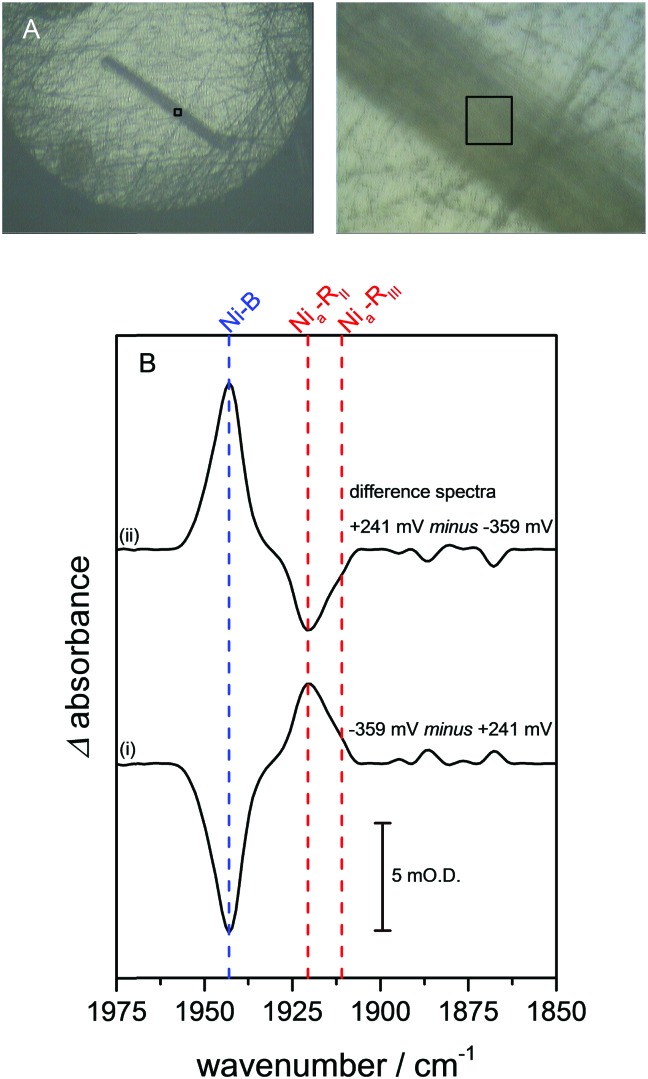
(A) Visible images, at 4× (left) and 36× (right) magnification, of a Hyd1 crystal deposited on the glassy carbon working electrode of the infrared microspectroscopic-electrochemical cell. A 15 × 15 μm^2^ sampling area, used to record the IR spectra presented in (B), is indicated with black squares. Images were recorded using the visible setting on a Bruker Hyperion 3000 microscope. In addition to the Hyd1 crystal these images show fine scratches that remain in the glassy carbon surface following the polishing procedure. (B) (i) Reduced *minus* oxidised difference spectrum following a step from +241 to –359 mV, and (ii) re-oxidised *minus* reduced spectrum following a step from –359 to +241 mV. Dashed lines show the wavenumber positions of the intrinsic *ν*_CO_ bands of the Ni–B (blue), Ni_a_–R_II_ and Ni_a_–R_III_ (red) states of the Hyd1 active site.^13^

Fig. 2B presents baseline-corrected IR difference spectra recorded at the 15 × 15 μm^2^ area on the Hyd1 crystal indicated in Fig. 2A, following (i) a reductive potential step from +241 mV to –359 mV and (ii) a subsequent re-oxidation step to +241 mV. The negative band in spectrum (i) at 1943 cm^–1^ corresponds to depletion of the oxidised inactive Ni–B state while positive bands indicate formation of the most reduced active site states, two forms of Ni_a_–R (Ni^II^(H–)Fe^II^, Scheme 1) at 1922 and 1914 cm^–1^, Ni_a_–R_II_ and Ni_a_–R_III_, previously characterised in Hyd1,^13^ and observed in a range of other NiFe hydrogenases.^10^–^12^ Additional spectral features are observed below 1900 cm^–1^, presumably corresponding to several Ni_a_–L states.^12^ Together, these spectra confirm that the electrochemical control over the crystal is completely reversible with potential. Spectral changes were complete within 30 minutes after the application of a potential step, and were reversible over multiple potential step sequences (not shown). Similar measurements were made at distinct positions along the same crystal (Fig. S2, ESI†), and show that the redox changes are reproduced along the crystal's length. The whole of the Ni–B peak intensity is lost upon reduction, and regained upon subsequent re-oxidation (Fig. S3, ESI†). The electrochemical method therefore provides redox control over the length and thickness of the whole single crystal.

Visible images recorded of the crystal over a period of several hours show no noticeable dissolution or change in dimension throughout the measurement (Fig. S4, ESI†). The crystals are stable at room temperature in the crystallisation buffer electrolyte solution under electrochemical control and exposed to mid-IR synchrotron radiation.

In previous studies of NiFe hydrogenases, multiple Ni_a_–R sub-states have been observed in response to reduction by either H_2_ or an electrode.^10^–^12^,^20^ The relative populations of the Ni_a_–R sub-states are constant over the entire potential range in which they are observed, implying that they are in rapid equilibrium with each other. The relative populations depend on the solution pH, suggesting that they differ in the location of a proton close to the NiFe active site.^20^ Time-dependent spectra for the Hyd1 crystal are shown in Fig. 3, following a reductive potential step from +241 mV to –359 mV, and reveal that for the first time the rates of formation of the Ni_a_–R_II_ and Ni_a_–R_III_ sub-states can be resolved (Fig. S5, ESI†). Establishment of the equilibrium between Ni_a_–R sub-states is retarded in the crystal relative to the situation in previous studies.^10^,^13^,^15^,^20^ This suggests that further studies on crystalline samples of Hyd1 should provide insight into sequential involvement of Ni_a_–R sub-states in the hydrogenase catalytic cycle.^12^ Similar changes were reproduced in analogous measurements using other Hyd1 crystals.

**Fig. 3:**
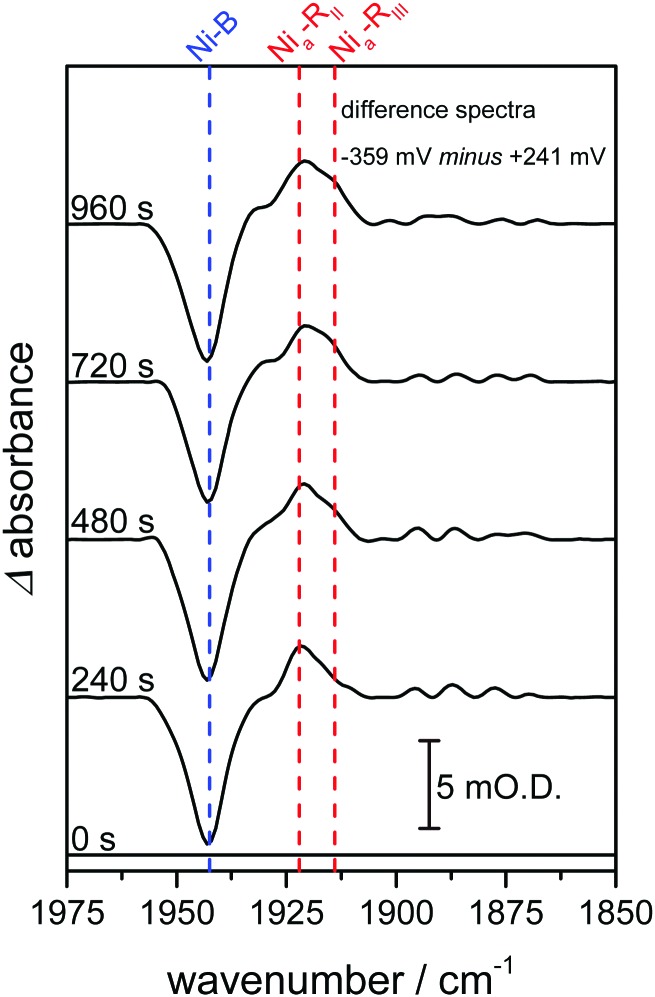
Infrared spectra recorded as a function of time highlight time-dependent formation of two Ni_a_–R states, Ni_a_–R_II_ and Ni_a_–R_III_. Spectra are presented as reduced minus oxidised baseline-corrected difference spectra following application of a reducing potential step from +241 to –359 mV and were recorded on the same Hyd1 crystal, and at the same position, as indicated in Fig. 2A.

Infrared microspectroscopy is an intrinsically non-invasive and non-destructive technique. In contrast, the high-powered laser sources required for related Raman microspectroscopy measurements can cause thermal damage to protein crystals or induce unwanted photochemistry that alters the state of the protein, as observed in a study on another NiFe hydrogenase.^29^ Also in contrast to Raman microspectroscopy, which typically only samples molecules close to the surface of a crystal, the IR approach we exploit here samples the entire thickness of the crystal. Therefore, IR microspectroscopy imaging is likely to be particularly useful in determining the redox state of non-haem metalloproteins in single crystals where the UV-visible or fluorescence spectroscopic changes are often weak or broad and difficult to interpret. This will allow direct monitoring of changes in ligand coordination or activation at metal centres of these enzymes.

The data presented here show that hydrogenases can be manipulated rapidly within a single crystal into different redox states, with confirmation of the states present at each potential provided by comparison of IR microspectroscopy data with IR spectra recorded for the same enzyme in solution or on an electrode. The fact that distinct kinetics of formation of two Ni_a_–R states are observed in the crystalline Hyd1 sample provides the interesting possibility of resolving chemical steps in crystalline protein samples that are otherwise too fast to resolve in solution.^5^,^30^ Intriguingly, it should also be possible to verify the redox state of the protein both before and after X-ray structure determination and, additionally, to generate specific redox states of a protein crystal for subsequent X-ray structural determination.

The work of FAA, SBC, RME and KAV is supported by BBSRC grants BB/L009722/1 and BB/N006321/1. Work of KAV, PAA, HAR and JSR is additionally supported by EPSRC grant EP/N013514/1. FAA is a Royal Society Wolfson Research Merit Award holder. AS is grateful for a scholarship from the Oxford Interdisciplinary Bioscience Doctoral Training Partnership, BB/M011224/1. We thank Diamond Light Source for access to the MIRIAM beamline B22 (SM13879) that contributed to the results presented here. We also thank Elena Nomerotskaia for assistance with isolation of Hyd1.

## Supplementary Material

Supplementary information

## References

[cit1] Bowman S. E. J., Bridwell-Rabb J., Drennan C. L. (2016). Acc. Chem. Res..

[cit2] Yano J., Kern J., Irrgang K.-D., Latimer M. J., Bergmann U., Glatzel P., Pushkar Y., Biesiadka J., Loll B., Sauer K., Messinger J., Zouni A., Yachandra V. K. (2005). Proc. Natl. Acad. Sci. U. S. A..

[cit3] Garman E. (2010). Acta Crystallogr., Sect. D: Biol. Crystallogr..

[cit4] Pearson A. R., von Stetten D., Huse N. (2015). Synchrotron Radiat. News.

[cit5] von Stetten D., Giraud T., Carpentier P., Sever F., Terrien M., Dobias F., Juers D. H., Flot D., Mueller-Dieckmann C., Leonard G. A., de Sanctis D., Royant A. (2015). Acta Crystallogr., Sect. D: Biol. Crystallogr..

[cit6] Evans R. M., Brooke E. J., Wehlin S. A. M., Nomerotskaia E., Sargent F., Carr S. B., Phillips S. E. V., Armstrong F. A. (2016). Nat. Chem. Biol..

[cit7] Evans R. M., Parkin A., Roessler M. M., Murphy B. J., Adamson H., Lukey M. J., Sargent F., Volbeda A., Fontecilla-Camps J. C., Armstrong F. A. (2013). J. Am. Chem. Soc..

[cit8] Krishnan S., Armstrong F. A. (2012). Chem. Sci..

[cit9] Reeve H. A., Lauterbach L., Lenz O., Vincent K. A. (2015). ChemCatChem.

[cit10] Lubitz W., Ogata H., Rüdiger O., Reijerse E. (2014). Chem. Rev..

[cit11] Shafaat H. S., Rüdiger O., Ogata H., Lubitz W. (2013). Biochim. Biophys. Acta, Bioenerg..

[cit12] Ash P. A., Hidalgo R., Vincent K. A. (2017). ACS Catal..

[cit13] Hidalgo R., Ash P. A., Healy A. J., Vincent K. A. (2015). Angew. Chem., Int. Ed..

[cit14] Ash P. A., Liu J., Coutard N., Heidary N., Horch M., Gudim I., Simler T., Zebger I., Lenz O., Vincent K. A. (2015). J. Phys. Chem. B.

[cit15] Murphy B. J., Hidalgo R., Roessler M. M., Evans R. M., Ash P. A., Myers W. K., Vincent K. A., Armstrong F. A. (2015). J. Am. Chem. Soc..

[cit16] Kurkin S., George S. J., Thorneley R. N. F., Albracht S. P. J. (2004). Biochemistry.

[cit17] Ogata H., Krämer T., Wang H., Schilter D., Pelmenschikov V., van Gastel M., Neese F., Rauchfuss T. B., Gee L. B., Scott A. D., Yoda Y., Tanaka Y., Lubitz W., Cramer S. P. (2015). Nat. Commun..

[cit18] Brecht M., van Gastel M., Buhrke T., Friedrich B., Lubitz W. (2003). J. Am. Chem. Soc..

[cit19] George S. J., Kurkin S., Thorneley R. N. F., Albracht S. P. J. (2004). Biochemistry.

[cit20] De Lacey A. L., Fernández V. M., Rousset M., Cammack R. (2007). Chem. Rev..

[cit21] Zhou Z.-Y., Lin S.-C., Chen S.-P., Sun S.-G. (2005). Electrochem. Commun..

[cit22] Zhou Z.-Y., Sun S.-G. (2005). Electrochim. Acta.

[cit23] Rosendahl S. M., Borondics F., May T. E., Pedersen T. M., Burgess I. J. (2011). Rev. Sci. Instrum..

[cit24] Rosendahl S. M., Borondics F., May T. E., Burgess I. J. (2013). Anal. Chem..

[cit25] Ash P. A., Reeve H. A., Quinson J., Hidalgo R., Zhu T., McPherson I. J., Chung M.-W., Healy A. J., Nayak S., Lonsdale T. H., Wehbe K., Kelley C. S., Frogley M. D., Cinque G., Vincent K. A. (2016). Anal. Chem..

[cit26] Bard A. J. and Faulkner L. R., Electrochemical Methods: Fundamentals and Applications, Wiley, New York, 2nd edn, 2001.

[cit27] Volbeda A., Martin L., Barbier E., Gutiérrez-Sanz O., De Lacey A. L., Liebgott P.-P., Dementin S., Rousset M., Fontecilla-Camps J. C. (2015). J. Biol. Inorg. Chem..

[cit28] Roessler M. M., Evans R. M., Davies R. A., Harmer J., Armstrong F. A. (2012). J. Am. Chem. Soc..

[cit29] Siebert E., Rippers Y., Frielingsdorf S., Fritsch J., Schmidt A., Kalms J., Katz S., Lenz O., Scheerer P., Paasche L., Pelmenschikov V., Kuhlmann U., Mroginski M. A., Zebger I., Hildebrandt P. (2015). J. Phys. Chem. B.

[cit30] Cavazza C., Bochot C., Rousselot-Pailley P., Carpentier P., Cherrier M. V., Martin L., Marchi-Delapierre C., Fontecilla-Camps J. C., Ménage S. (2010). Nat. Chem..

