# Supplementary material for: Generating single metalloprotein crystals in well-defined redox states: electrochemical control combined with infrared imaging of a NiFe hydrogenase crystal
Source: Chem Commun (Camb). 2017 May 9;53(43):5858–61. doi: 10.1039/c7cc02591b (PMC5708527; doi:10.1039/c7cc02591b)
Supplement: Supplementary file 1 [file CC-053-C7CC02591B-s001.pdf]

Electronic Supplementary Material (ESI) for Chemical Communications.
This journal is © The Royal Society of Chemistry 2017

Electronic Supplementary Information

Generating single metalloprotein crystals in well-defined redox states: electrochemical control combined with infrared imaging of a NiFe hydrogenase crystal

P. A. Ash,[a] S. B. Carr,[b,c] H. A. Reeve,[a] A. Skorupskaitė,[a] J. S. Rowbotham,[a] R. Shutt,[a] M. D. Frogley,[d] R. M. Evans,[a] G. Cinque,[d] F. A. Armstrong,[a] K. A. Vincent[a,*]

[a] Department of Chemistry, University of Oxford, Inorganic Chemistry Laboratory, South Parks Road, Oxford, OX1 3QR, UK.

[b] Research Complex at Harwell, Rutherford Appleton Laboratory, Didcot, Oxfordshire OX11 0FA, UK.

[c] Department of Biochemistry, University of Oxford, South Parks Road, Oxford, OX1 3QU

[d] Diamond Light Source, Harwell Science and Innovation Campus, Didcot, Oxfordshire OX11 0QX, UK

* Corresponding author email address: kylie.vincent@chem.ox.ac.uk

Hyd1 Crystal Preparation

Wild type Hyd1 (full length protein including the C-terminal transmembrane helix) was purified as described previously and concentrated to 5 mg mL^{-1}.[1] Crystals were grown aerobically using the sitting drop vapour diffusion technique, whereby 1 μL of protein solution was mixed with an equal volume of crystallisation buffer (100 mM Bis-tris pH 6.0, 150 mM NaCl, 200 mM Li_2SO_4, 20-23 wt.% PEG 3350) and allowed to equilibrate against a 500 μL reservoir of the same. X-ray diffraction data have been collected from other crystals grown from the same batch of protein (but not the crystals used for IR measurements presented here). Crystals from this batch diffracted x-rays to a resolution of 1.2 Å.

Figure S1. Cyclic voltammogram demonstrating electrochemical control within the infrared microspectroscopic-electrochemical cell. Recorded in the presence of a Hyd1 crystal at a scan rate of 10 mV/s, with a background electrolyte of crystallisation buffer (100 mM Bis-tris pH 6.0, 150 mM NaCl, 200 mM Li_2SO_4, 20-23 wt.% PEG 3350) additionally containing the redox mediators potassium ferricyanide, methylene blue, 2-hydroxy-1,4-naphthoquinone and methyl viologen (at a concentration of ca 1 mM each). The mid-point potentials for each mediator are relatively consistent with values reported in the literature (Table S1) showing that there is no significant potential offset within the cell, although the cause of the large peak-to-peak separation in the case of 2-hydroxy-1,4-naphthoquinone is unclear. For the infrared spectroscopic data reported in the main text we chose to omit potassium ferricyanide due to the similar wavenumber positions of the ν_{CN} bands of ferri/ferrocyanide and intrinsic active site bands of Hyd1.

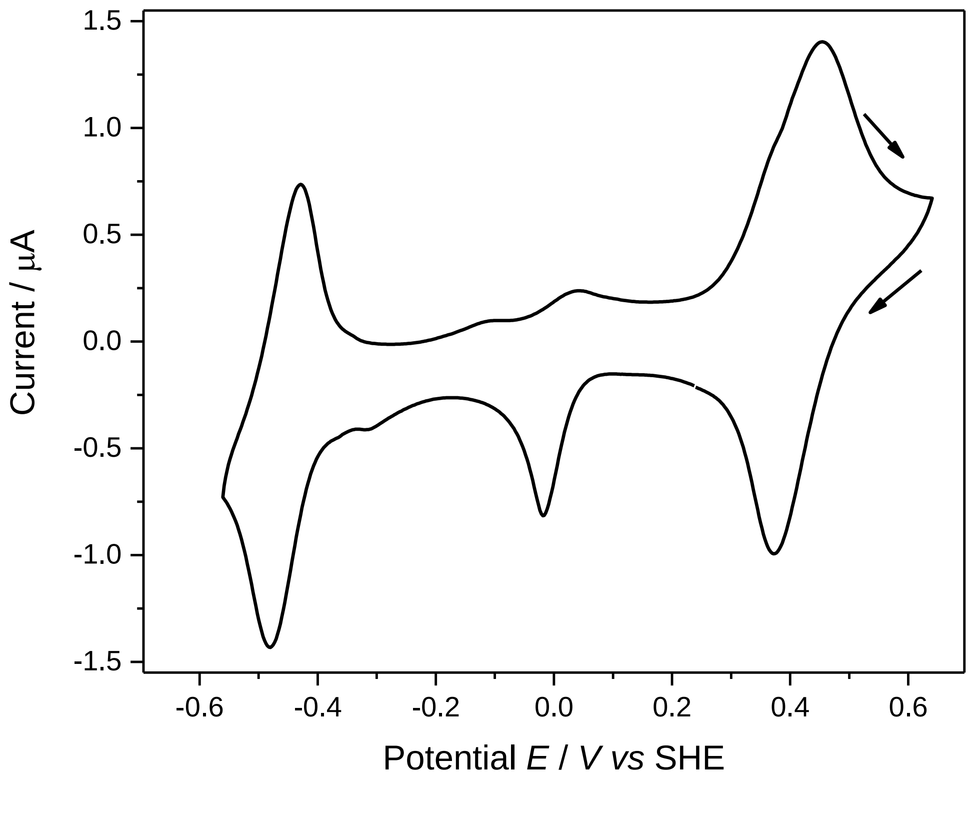

Table S1. Mid-point potentials (E_m) and peak-to-peak separations (ΔE_p) of redox mediators used in this work, measured in the infrared microspectroscopic-electrochemical cell. Literature values of E_m are provided for comparison.

Redox Mediator	Measured E_m / mV[(a)]	Literature E_m / mV[(b)]	ΔE_p / mV
Potassium ferricyanide	+413	+430[2,3]	83
Methylene blue	+12	+11[3–5]	61
2-hydroxy-1,4-naphthoquinone	−209	−139[6,7]	225
Methyl viologen	−455	−446[3,5,8]	51

(a) Measured a pH 6.0 in crystallisation buffer. (b) Reported at pH 7.

Figure S2. Difference spectra recorded at various points along a single Hyd1 crystal demonstrate the uniformity of potential-induced changes throughout the crystal. Panel A shows a visible image of the same single Hyd1 crystal used to record the difference spectra presented in Figures 2 and 3 of the main text, showing the location of four 15 × 15 μm^2 areas at which the difference spectra shown in Panel B were recorded (the numbering reflects the order of data collection). To record each difference spectrum in Panel B the Hyd1 crystal was equilibrated at a potential of +241 mV before application of a potential of −359 mV for 30 minutes. The general features in each of the difference spectra are very similar, demonstrating the whole crystal sample responds to the applied potentials.

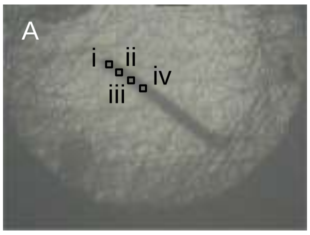

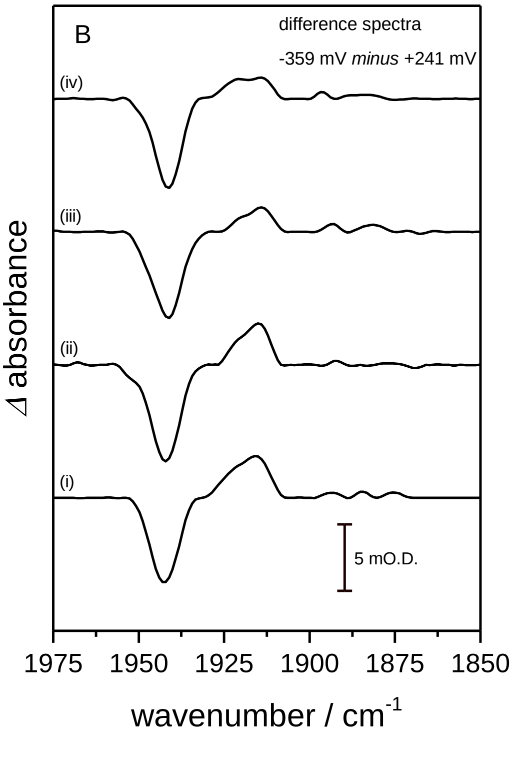

Figure S3. All of the initial Ni-B intensity is lost upon reduction, and regained upon subsequent re-oxidation. A baseline corrected absolute absorbance spectrum of the Hyd1 crystal, in the initial oxidised Ni-B state, at the position indicated in Figure 2A of the main manuscript (black, relative to a portion of the working electrode free from protein crystal) compared to the re-oxidised *minus* reduced difference spectrum (red, reproduced from Figure 2B(ii) of the main manuscript). Figure 2B demonstrated that the redox changes are fully reversible. Since the absolute magnitude of the Ni-B peak in the difference spectra is equal to the absolute magnitude of the initial Ni-B peak through the whole thickness of the crystal, all active sites within the crystal sample respond to the applied potential.

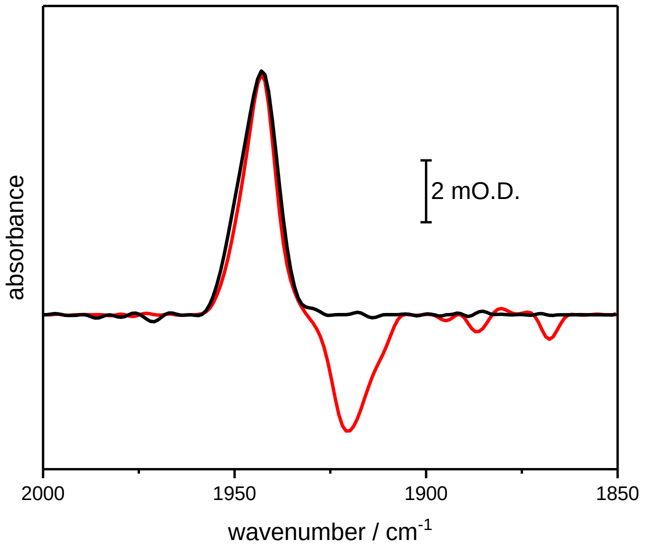

Figure S4. Visible images of a single crystal, recorded at 36× magnification, show no significant change in appearance or size of the crystal over the course of 14 hours of measurements. The image in Panel A was recorded immediately prior to reductive activation of the Hyd1 crystal, in preparation for collection of all spectroscopic data presented in the main manuscript. Panels B-D show visible images at various points along the same crystal, recorded immediately after collection of the position-dependent difference spectra shown in Figure S2. Note that Panels A and C show approximately the same position on the crystal; a distinctive ‘cross’-shaped scratch on the glassy carbon electrode surface is marked with dashed lines in Panels A, C and D as a guide to the relative positions of each image. The black squares represent a 15 × 15 µm^2 area.

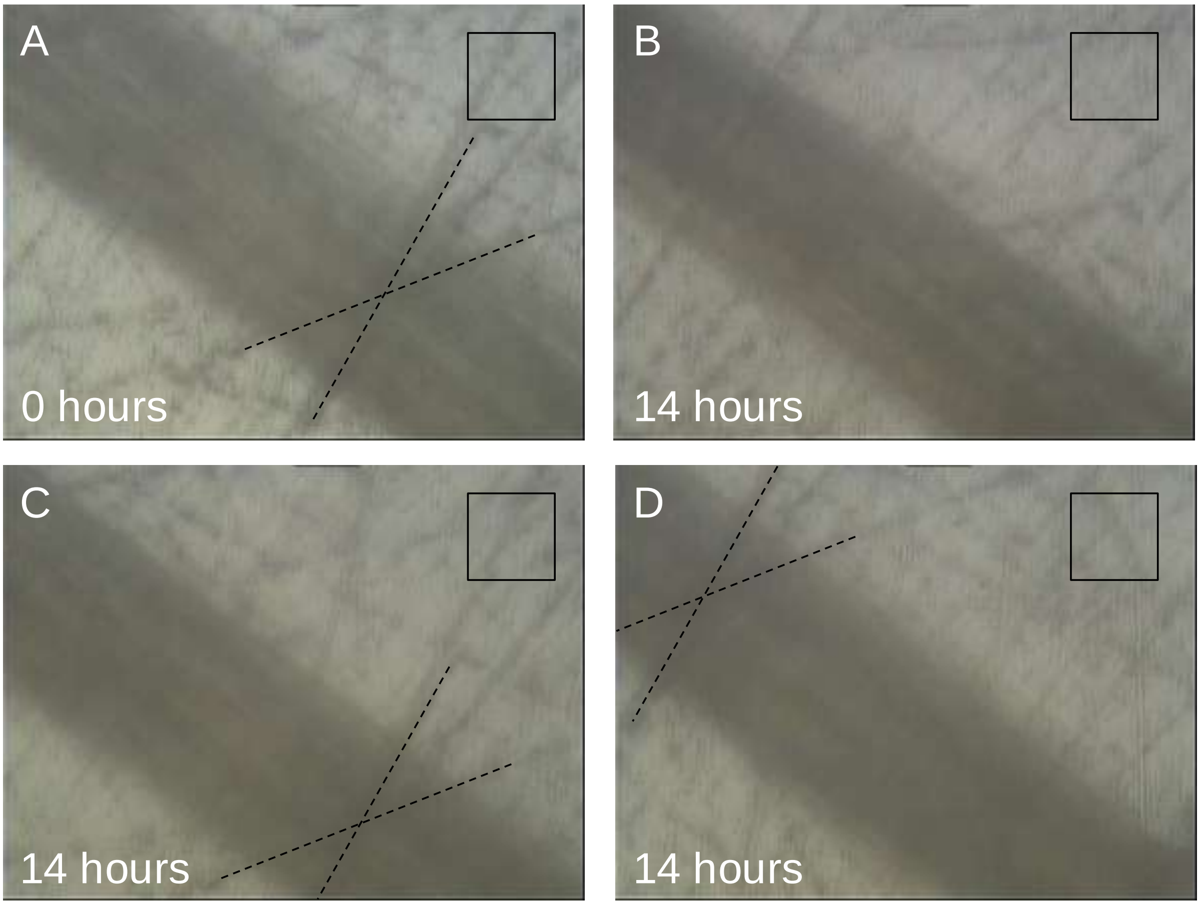

Figure S5. Time-dependent formation of the Ni_a-R_{II} and Ni_a-R_{III} states following a reductive potential step from +241 mV to −359 mV. Data were extracted from the difference spectra in Figure 3 of the main text and show the change in absorbance at 1922 cm^{-1} (Ni_a-R_{II}) and 1914 cm^{-1} (Ni_a-R_{III}) as a function of time. The peak absorbances were extracted by fitting multiple Gaussian curves to the difference spectra in Figure 3.

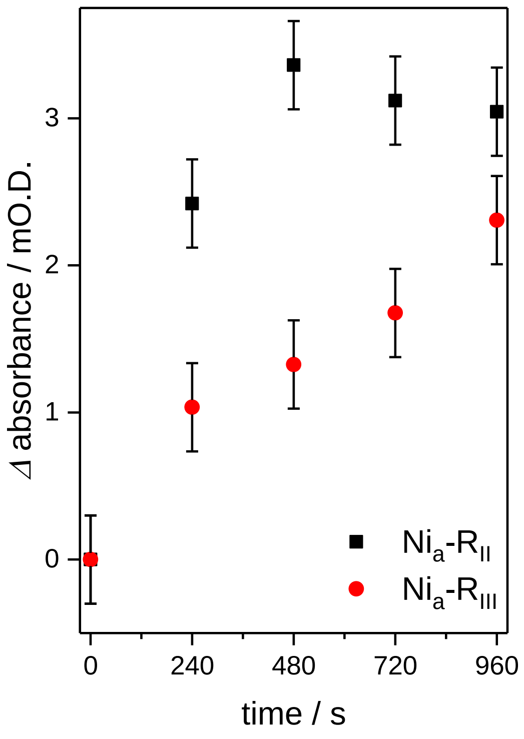

References

1 M. J. Lukey, A. Parkin, M. M. Roessler, B. J. Murphy, J. Harmer, T. Palmer, F. Sargent and F. A. Armstrong, *J. Biol. Chem.*, 2010, **285**, 3928–3938.

2 I. M. Kolthoff and W. J. Tomsicek, *J. Phys. Chem.*, 1934, **39**, 945–954.

3 R. Cammack, V. M. Fernandez and E. Claude Hatchikian, *Methods Enzymol.*, 1994, **243**, 43–68.

4 P. S. Rao and E. Hayon, *J. Phys. Chem.*, 1973, **77**, 2753–2756.

5 W. N. Lanzilotta and L. C. Seefeldt, *Biochemistry*, 1997, **36**, 12976–12983.

6 L. Nedbal, G. Samson and J. Whitmarsh, *Proc. Natl. Acad. Sci.*, 1992, **89**, 7929–7933.

7 B. Bleijlevens, F. A. van Broekhuizen, A. L. D. Lacey, W. Roseboom, V. M. Fernandez and S. P. J. Albracht, *J. Biol. Inorg. Chem.*, 2004, **9**, 743–752.

8 C. L. Bird and A. T. Kuhn, *Chem. Soc. Rev.*, 1981, **10**, 49–82.
